# Prevalence of Anemia and Associated Factors among Pregnant Women in an Urban Area of Eastern Ethiopia

**DOI:** 10.1155/2014/561567

**Published:** 2014-08-25

**Authors:** Kefyalew Addis Alene, Abdulahi Mohamed Dohe

**Affiliations:** ^1^Institute of Public Health, College of Medicine and Health Sciences, University of Gondar, P.O. Box 196, Gondar, Ethiopia; ^2^Somali Regional Health Bureau, P.O. Box 238, Jijiga, Ethiopia

## Abstract

This research work presents the magnitude of anemia and its determinant factors among pregnant women. As far as this research is done in the eastern part of Ethiopia, where there is a different cultural issue related to pregnancy and dietary habit, it will help the researchers to know the problem in different parts of the country.

## 1. Background 

Anemia, defined as a decreased concentration of blood hemoglobin, is one of the most common nutritional deficiency diseases observed globally and affects more than a quarter of the world's population [1–8]. It is a major public health problem affecting all ages of the population with its highest prevalence among children under five years of age and pregnant women [2, 3]. Globally, anemia affects 1.62 billion people (25%), among which 56 million are pregnant women [1, 2].

Anemia during pregnancy is considered severe when hemoglobin concentration is less than 7.0 g/dL, moderate when hemoglobin falls between 7.0–9.9 g/dL, and mild from 10.0-11 g/dL [2–4]. Anemia during pregnancy is a major cause of morbidity and mortality of pregnant women in developing countries and has both maternal and fetal consequences [9–13]. It is estimated that anemia causes more than 115,000 maternal and 591,000 perinatal deaths globally per year [3].

In developing countries, the cause of anemia during pregnancy is multifactorial and includes nutritional deficiencies of iron, folate, and vitamin B12 and also parasitic diseases, such as malaria and hookworm. The relative contribution of each of these factors to anemia during pregnancy varies greatly by geographical location, season, and dietary practice. In Sub-Saharan Africa, iron and folate deficiencies are the most common causes of anemia in pregnant women [14]. Anemia has a variety of converging contributing factors including nutritional, genetic, and infectious disease factors; however, iron deficiency is the cause of 75% of anemia cases [2, 5, 8–15]. Iron deficiency anemia affects the development of the nation by decreasing the cognitive development of children and productivity of adults [2, 10].

Seventeen percent of Ethiopian women in the reproductive age group are anemic and 22% of these women are currently pregnant [16]. Despite its known effect on the population, there is very little data available in the study area. Therefore, this study is aimed at determining the prevalence of anemia in pregnant women and identifying its associated factors in the Somali Region of Eastern Ethiopia.

## 2. Methods

### 2.1. Study Setting and Design

A community based cross-sectional study was conducted from April to May 2013 in Gode town, Eastern Ethiopia.

### 2.2. Study Population

The study population consisted of a sample of pregnant women who were residing in the town during the study period. Those pregnant women who were not long-term residents of the city (less than 6 months) were excluded.

### 2.3. Sampling Procedures

Sample size was determined based on single population proportion formula using Epi info version 7 with a 95% CI, 5% margin of error, and assumption that 22% of pregnant women are anemic [16]. Assuming a 10% nonresponse rate and a design effect of 2, a total sample size of 581 pregnant women was required. Multistage sampling technique was used to select the study participants. Four kebeles (villages) were selected from 6 kebeles using the randomized method. A proportional allocation was employed to obtain the sample size from each kebele. The starting point was randomly selected and a systematic random sampling method was used to select the study participants.

### 2.4. Data Collection

Data were collected using pretested interviewer administered questionnaire, which contains sociodemographic characteristics (age, education, occupation, marital status, and others), obstetric and gynecological history (trimester, gravidity, parity, ANC follow up, iron supplementation, and others), and dietary factors (type of stable diet, meal frequency, and intake of meat, tea, egg, and milk and milk products).

Blood hemoglobin concentration was measured using a HemoCue Hb 301, a precalibrated instrument designed for the measurement of hemoglobin concentration. Venous blood was drawn, through microcuvettes, and inserted into the HemoCue and the result was recorded. Mid upper arm circumference (MUAC) of the mothers was measured and recorded as an independent variable.

### 2.5. Data Processing and Analyses

Data were analyzed using SPSS version 20. Description of means, frequencies, proportions, and rates of the given data for each variable was calculated. Bivariate analysis was done to see the association of each independent variable with the outcome variable. Those variables having *P* value less than 0.2 were entered into the multivariate logistic regression model to identify the effect of each independent variable with the outcome variables. A *P* value of less than 0.05 was considered statistically significant, and adjusted odds ratio with 95% CI was calculated to determine association.

### 2.6. Ethical Consideration

Ethical clearance was obtained from the Institutional Review Board of the University of Gondar. An official letter was obtained for Somali Regional Health Bureau and Gode City Administration Health Office and letters were prepared for the local authority of the selected kebeles. Written informed consent was obtained from each study participant after they were introduced to the purpose of the study and informed about their rights to interrupt the interview at any time. Confidentiality was maintained at all levels of the study. Mothers found to be anemic were referred to the nearest health center or hospital and the referral process was facilitated.

## 3. Results

### 3.1. Socioeconomic and Demographic Characteristics

Out of a total sample size (581) 577 pregnant women were included and there was a nonresponse rate of 0.7%. The mean ages of the mothers at present, at marriage, and at first pregnancy were 27.01 (±5.97), 17.52 (±2.10), and 18.38 (±2.32) years, respectively. Majority of the mothers were married (97.2%), Somali (98.9%), and Muslim (99.1%).

The average family size, number of pregnancies, deliveries, and children of the households were 6.9 (±2.86), 4.98 (±2.62), 3.83 (±2.55), and 3.61 (±2.38), respectively. About 261 (45.2%) of the mothers were in their second trimester, 247 (24.8%) were in the third trimester, and the remaining 69 (12.0%) were in the first trimester.

Most of the mothers were illiterate 507 (87.9%) and housewives 499 (86.5%). The family's main sources of income were salaried jobs 301 (52.2%), private business 101 (17.5%), and remittance 52 (9%). More than half of the respondents 355 (61.5%) were at or below the middle wealth quintile (Table 1).

### 3.2. History of Malaria and Intestinal Parasite

Of the 577 respondents, 88 (15.3%) had a known history of intestinal parasite, 548 (95.0%) said they were using insecticide treated bed net (ITNs), and 366 (63.4%) had a known history of malaria. Of those with a known history of intestinal parasite 7 (8%) were infected during the current pregnancy, 39 (44.3%) were infected 1–3 months before the current pregnancy, and the remaining 42 (47.8%) were infected more than four months prior to the current pregnancy. Similarly, from those with known history of malaria 37 (10.1%) were infected during the current pregnancy, 131 (35.8%) were infected 1–3 months before current pregnancy, and the remaining 198 (54.1%) were infected more than four months before current pregnancy.

### 3.3. Environmental and Housing Characteristics of the Study Subjects

Of the 577 respondents 211 (36.6%) were living in a rented house, and the remaining 366 (63.4%) owned the house. Four hundred fifty one (78.2%) had private latrine, but only 26 (4.5%) had piped water. Only 18 (3.1%) had tap water, and only 2 (0.3%) had public tap water as their main source of drinking water. Half (56.5%) of the study participants obtained drinking water from a protected well/tanker and the remaining 231 (40%) drank water from arriver. Also, only 34 (5.9%) of the respondents had a farm or garden to cultivate and assist them in meeting their nutritional needs.

### 3.4. Behavioral Characteristics and Nutritional Status of Respondents

The most reported staple diet of the household was rice and spaghetti 504 (87.3%). More than half, 382 (66.2%), of the respondents eat three times per day.

The majority of the respondents 570 (98.8%) said they drink tea and 444 (77.9%) of them drink tea before meals, and 339 (59.5%) of them have tea more than two times per day. Five hundred fifty four (96%) and 384 (66.6%) had eaten meat and used fruits more than twice per week, respectively. More than half of the respondents, 348 (60.3%), said they use egg less than once per month, but 312 (54.1%) use milk and milk products daily, and 416 (72.1%) had eaten between 5 and 8 food groups in the last 24 hours prior to the interview.

Of the 577 respondents 177 (30.7%) reported involvement in physical work with 27 (15.3%) and 30 (16.9%) of them reporting very heavy and heavy physical work, respectively. One hundred seven (18.5%) reported consuming forbidden foods and 196 (34%) have reported eating charcoal or clay during pregnancy. More than half of 347 (60.1%) said they had visited a health facility for antenatal care (ANC) with 56 (16.1%), 116 (33.4%), and 175 (50.4%) of them reporting an initial ANC visit, a second ANC visit, and more than two ANC visits during a pregnancy. More than half of 388 (67.2%) reported receiving iron supplementation during the current pregnancy.

Nutritional status was evaluated by MUAC and 575 of the respondents were measured (excluding 2 with incomplete MUAC), and 29 (5%) had MUAC of less than 21 cm, 228 (39.7%) had MUAC between 21 and 23 cm, and the remaining 318 (55.3%) had an MUAC within normal limits (>23 cm) (Table 2).

### 3.5. Prevalence of Anemia

From the total 577 respondents 328 (56.8%) were anemic (95% CI: 52.9–60.8), 7 (1.2%) of them were severely anemic, 154 (26.7%) were moderately anemic, and 167 (28.9%) were mildly anemic. The mean hemoglobin level was 10.79 (±1.47) g/dL. More than half (76.8%) of the anemic study participants were from large families (>5 family members). Most of them (80.7%) were multiparous (≥2) and multigravidous (≥3). Majorities were married at age 18 years or younger (82.9%). Most of them (58.5%) were at or below the middle wealth quintile and were illiterate (89.9%).

### 3.6. Factors Associated with Anemia

Multivariate logistic regression analysis revealed that trimester of current pregnancy, iron supplementation during pregnancy, wealth quintile, number of pregnancies, and MUAC were significantly associated with anemia in the study population (Table 4).

Pregnant women in the third and second trimesters were 3.32 (95% CI 1.84–6.0) and 2.87 (95% CI 1.61–5.17) times more likely to be affected by anemia as compared to pregnant women in the first trimester, respectively. Similarly, those pregnant women who had 3–5 pregnancies were 1.95 times more likely to be anemic, compared with those who had less than 3 pregnancies (AOR =1.95 (95% CI=1.19–3.19)).

Pregnant women who were not taking iron supplementation during pregnancy were 1.54 more likely to develop anemia (AOR = 1.54 (95% CI = 1.04–2.27)).

Pregnant women in the second wealth quintile were 57% (AOR = 0.43, 95% CI: 0.24–0.76) less likely to develop anemia, compared with those in the lowest wealth quintile. Similarly, pregnant women with MUAC ≥ 23 were 59% less likely to be anemic compared with those with MUAC < 23 (AOR = 0.41 (95% CI: 0.27–0.63)) (Table 3).

## 4. Discussion

More than half (56.8%) of the pregnant women studied were anemic. This figure is lower than the prevalence of anemia in pregnant women of some other developing countries such as India and Pakistan [4, 15]. This may be due to the inclusion of more rural villages in the studies from Pakistan and India, which were not included in this study. This finding is much higher than the national prevalence of anemia in pregnant women, which is 22% [16], and also higher than the prevalence noted from the 2011 Ethiopian Demographic Health Survey (EDHS) report, which found that 30.4% of Ethiopians were anemic [8]. This discrepancy could be due to the exclusion of agropastoralist zones and the time gap between the current study and the 2011 EDHS.

Gravidity and age of current pregnancy (trimester) were important variables, which have shown a significant association with anemia in the current study. The risk of developing anemia increases with the age of pregnancy (trimester). The risk of developing anemia was higher in third and second trimester when compared with those in the first trimester. This finding is consistent with a study done in Saudi Arabia, which found that the prevalence of anemia is higher in the third trimester in comparison with first trimester [11], and another study conducted in India, which also indicated that the prevalence of anemia was higher in pregnant women in the third and second trimesters [10]. Additionally, studies conducted in Malaysia, Vietnam, and Nepal found that increased gestational age is significantly associated with the risk of developing anemia [6, 17, 18]. This could be due to the fact that when the gestational age increases the mother becomes weak and the iron in the blood is shared with the fetus in the womb therefore decreasing the iron binding capacity of the mother's blood.

The other important variable significantly associated with anemia is number of pregnancies (gravidity). The risk of developing anemia in pregnant women with 3–5 pregnancies is increased when compared with those who had less than 3 pregnancies. This finding is consistent with studies conducted in Saudi Arabia and India, which found that increased number of pregnancies and deliveries is positively associated with the risk of developing anemia [10, 11]. This could be due to the loss of iron and other nutrients during increased and repeated pregnancies and also the possibility of sharing of resources with the fetus. However, other studies conducted in Ethiopia and Nepal did not find association between gravidity and anemia [8, 18]. This could be due to the difference in sociocultural characteristics of the study populations.

Similarly the wealth status of the household was significantly associated with the development of anemia. Increased wealth quintiles from lowest to second lowest reduce the risk of developing anemia by 57%. This finding is consistent with the findings from other studies in low and middle-income countries, which found that the risk of anemia among women living in the lowest wealth quintile was increased when compared with those living in the fourth and fifth wealth quintiles. A study done in India showed that pregnant women from lower socioeconomic classes were at increased risk of developing anemia compared with those in higher socioeconomic classes [2, 10]. This could be due to the fact that those from lower socioeconomic status (SES) lack the ability to purchase the quality or quantity of foods compared with those from higher SES.

Many other studies on anemia in pregnancy conducted in Ethiopia, Pakistan, Nepal, Vietnam, and Malaysia [5, 6, 8, 17, 18] did not consider the effect of wealth index. This could be because the wealth index is calculated from different household assets.

Two other variables with significant association with anemia were iron supplementation during pregnancy and MUAC level. The risk of developing anemia increased in pregnant women who did not receive iron supplementation during pregnancy when compared with those who received iron supplementation. This finding is consistent with the findings from studies in Vietnam and India, which indicated that lack of iron supplementation is among the most significant risk factors for developing anemia during pregnancy [6, 10]. This is likely due to the fact that the requirement of iron increases for pregnant women compared with nonpregnant women, and if they do not receive iron supplementation, the iron they ingest from food sources is not adequate to meet their needs. The finding contrasts with a study done at Pakistan, which found that women who reported consuming iron supplementation had a significantly lower hemoglobin concentration. This may be due to a difference in methodology between the studies.

MUAC less than 23 is found to increase the risk of developing anemia. The current study has shown that pregnant women with MUAC ≥ 23 had 59% less risk of developing anemia. This finding is consistent with a study done in Nepal, which found that MUAC > 23.6 significantly decreased risk of developing anemia [18]. This can be explained by the fact that undernourished pregnant women have a higher probability of being micronutrient deficient and therefore iron deficient and anemic.

Other independent variables which are not significant factors in this study but found to be significant by other studies reviewed include age of the mother, age at pregnancy, age at marriage, number of deliveries, number of children, family size, educational status of the mother, and occupation of the mother [5, 9, 14]. This could be due to the difference between sociocultural and behavioral characteristics of the community in this study and previous studies.

This study was conducted at the community level and considered altitude as an independent variable to assess anemia. Limitations include the cross-sectional nature of the study and using patient report rather than laboratory data for the identification of previous malarial and parasitic infections. Also, the study did not include rural residents.

## 5. Conclusion

The prevalence of anemia among pregnant women in this study was high compared with women in other areas of Ethiopia. Trimester of current pregnancy, wealth quintile, gravidity, iron supplementation, and MUAC were found to be significantly associated with anemia. Iron supplementation and special care during late pregnancy are recommended to reduce anemia. Further research on risk factors of anemia, which include rural residents, should be conducted to strengthen and broaden these findings.

## Figures and Tables

**Table 1:** Sociodemographic characteristics of pregnant women at Gode town, Somali Region, Eastern Ethiopia, May 2013.

Variables	Frequency (*N* = 577) Number (%)
Age of the mother	
≤20	62 (10.7)
21–25	137 (23.7)
26–30	187 (32.4)
31–35	122 (21.1)
>35	69 (12.0)
Marital status	
Married	561 (97.2)
Divorced	13 (2.3)
Widowed	3 (0.5)
Religion	
Muslim	572 (99.1)
Christian	5 (0.9)
Ethnicity	
Somali	570 (98.8)
Amhara	5 (0.9)
Oromo	1 (0.2)
Others	1 (0.2)
Educational status	
Unable to read and write	476 (82.5)
Read and write only	31 (5.4)
Primary school (1–8)	37 (6.4)
Secondary school	8 (1.4)
College and above	25 (4.3)
Occupation	
Housewife	499 (86.5)
Government employee	34 (5.9)
Business	44 (7.6)
Family Size	
<5	134 (23.2)
5–7	211 (36.6)
≥8	232 (40.2)
Age at marriage	
<16	198 (34.3)
16–18	283 (49.0)
≥19	96 (16.6)
Source of income	
Salary	301 (52.2)
Business	101 (17.5)
Remittance	52 (9.0)
Others	123 (21.3)
Wealth index (Quintiles)	
Lowest	114 (19.8)
Second	127 (22.0)
Middle	114 (19.8)
Fourth	104 (18.0)
Highest	118 (20.5)

**Table 2:** Maternal characteristics of pregnant women at Gode Town, Somali Region, Eastern Ethiopia, May 2013.

Variables	Frequency (*N* = 577) Number (%)
Trimester	
First trimester	69 (12.0)
Second trimester	261 (45.2)
Third trimester	247 (42.8)
Age at first pregnancy (years)	
<17	126 (21.8)
17–19	270 (46.8)
≥20	181 (31.4)
Number of pregnancies	
<3	166 (20.1)
3–5	228 (39.5)
≥6	233 (40.4)
Number of deliveries (parity)	
<2	121 (21.0)
2–4	237 (41.1)
≥5	219 (38)
Number of Children	
<2	126 (21.8)
2–4	247 (42.8)
≥5	204 (35.4)
Child spacing	
Yes	42 (7.3)
No	535 (92.7)
Antenatal care	
Yes	347 (60.1)
No	230 (39.9)
ANC during current pregnancy (*n* = 347)	
Yes	322 (92.8)
No	25 (7.2)
Frequency of ANC (347)	
First ANC	56 (16.1)
Second ANC	116 (33.4)
Third ANC	175 (50.4)

**Table 3:** Dietary habits and nutritional status of pregnant women at Gode Town, Somali Region, Eastern Ethiopia, May 2013.

Variables	Frequency (*N* = 577) Number (%)
Stable diet	
Rice and spaghetti	504 (87.3)
Maize and sorghum	68 (11.8)
Others	5 (0.9)
Meal frequency	
More than three times	120 (20.8)
Three times	382 (66.2)
Two times	63 (10.9)
One time	12 (2.1)
Meat frequency	
One a week	137 (23.7)
Twice a week	267 (46.3)
More than twice per week	150 (26)
Once per month	7 (1.2)
Less than one time per month	16 (2.8)
Drinking tea	
Yes	570 (98.8)
No	7 (1.2)
Time for drinking tea (*N* = 570)	
Before meal	444 (77.9)
After Meal	126 (22.1)
Fruit frequency	
Every day	28 (4.9)
Once a week	67 (11.6)
Twice a week	178 (30.8)
More than twice per week	111 (19.2)
Once per month	70 (12.1)
Less than once per month	348 (60.3)
Egg frequency	
Every day	25 (4.3)
Once a week	65 (11.3)
Twice a week	48 (8.3)
More than twice per week	21 (3.6)
Once per month	70 (12.1)
Less than once per month	348 (60.3)
Milk and milk product frequency	
More than two times per day	312 (54.1)
Once per day	160 (27.7)
Once per week	43 (7.5)
Less than once per week	62 (10.7)
Food groups eaten in 24 hours	
1–4	141 (24.4)
5–8	416 (72.1)
>8 food groups	20 (3.5)
Physical work	
Yes	177 (30.7)
No	400 (69.3)
Forbidden foods for pregnancy	
Yes	107 (18.5)
No	470 (81.5)
Eating charcoal and clay	
Yes	196 (34)
No	381 (66)
Iron supplementation	
Yes	388 (67.2)
No	189 (32.8)
Nutritional status (MUAC) (*n* = 575)	
<21 cm	29 (5)
21–23 cm	228 (39.7)
>23 cm	318 (55.3)

**Table 4:** Factors associated with anemia among pregnant women in Gode town, Somali Region, Eastern Ethiopia; May 2013.

Variables	Anemia status		COR (95% CI)	AOR (95% CI)
	Anemic	Normal		
Trimester				
First trimester	24	45	1.00	1.00
Second trimester	155	106	***2.74 (1.58–4.77)***	***2.87 (1.61–5.17)***
Third trimester	149	98	***2.85 (1.63–4.98)***	**3.32 (1.84–6.00)**
Parity				
<2	63	58	*1.00 *	
2–4	150	87	*1.59 (1.02–2.47) *	
≥5	115	104	*1.02 (0.65–1.59) *	
Family size				
<5	76	58	*1.00 *	
5–7	135	76	*1.29 (0.84–1.98) *	
≥8	117	115	*1.75 (1.19–2.56) *	
Forbidden foods				
Yes	70	37	*1.00 *	
No	258	212	*0.64 (0.41–0.99) *	
ANC				
Yes	184	163	*1.00 *	
No	144	86	*0.74 (0.52–1.05) *	
Iron supplementation during pregnancy				
Yes	207	181	1.00	1.00
No	121	68	0.72 (0.45–1.15)	**1.54 (1.04–2.27)**
24 hr recall food groups				
1–4	94	47	1.00	
5–8	224	192	0.58 (0.39–0.87)	
>8	10	10	0.50 (0.2–1.29)	
Wealth index				
Lowest	49	65	1.00	1.00
Second	78	49	0.40 (0.24–0.68)	**0.43 (0.24–0.76)**
Middle	65	49	0.85 (0.50–1.43)	0.989 (0.57–1.72)
Fourth	59	45	0.71 (0.42–1.20)	0.69 (0.39–1.21)
Highest	77	41	0.70 (0.41–1.20)	0.67 (0.37–1.19)
Gravidity				
<3	59	57	1.00	1.00
3–5	148	80	1.79 (1.14–2.82)	**1.95 (1.19–3.19)**
≥6	121	112	1.04 (0.67–1.63)	1.14 (0.7–1.85)
MUAC				
<23	107	44	1.00	1.00
≥23	219	205	0.44 (0.30–0.66)	**0.41 (0.27–0.63)**
